# Accuracy of Acceleration Time of Distal Arteries to Diagnose Severe Peripheral Arterial Disease

**DOI:** 10.3389/fcvm.2021.744354

**Published:** 2022-01-20

**Authors:** Jean-Eudes Trihan, Guillaume Mahé, Magali Croquette, Vicky Coutant, Cécile Thollot, Jérôme Guillaumat, Damien Lanéelle

**Affiliations:** ^1^Vascular Medicine Unit, University Hospital of Poitiers, Poitiers, France; ^2^Univ Rennes, M2S – EA 7470, Rennes, France; ^3^Vascular Medicine Unit, University Hospital Rennes, Rennes, France; ^4^Clinical Investigation Center, INSERM CIC 1414, Rennes, France; ^5^Vascular Medicine Unit, University Hospital Côte de Nacre, Caen, France; ^6^UNICAEN, INSERM 1075, COMETE, Caen, France

**Keywords:** peripheral arterial disease, Doppler ultrasound, acceleration time, critical limb ischemia, ankle-brachial index (ABI), toe-brachial index (TBI)

## Abstract

**Context:**

Ankle-brachial index (ABI) and toe-brachial index (TBI) are the recommended tests for the diagnosis of lower extremity peripheral artery disease (PAD) and the assessment of its severity, whereas Doppler ultrasound (DUS) is usually used to localize vascular lesions. However, the performance of DUS as an alternative to TBI and ABI measurement is unknown.

**Objective:**

The goals were (i) to evaluate the correlation between DUS parameters of distal arteries of the lower extremities with TBI in patients with PAD; (ii) to evaluate the correlation between DUS parameters of distal arteries with ABI; and (iii) to assess the diagnostic accuracy of maximal acceleration time of pedal arteries to detect toe pressure ≤30 mmHg.

**Methods:**

An observational retrospective study was conducted for 1 year on patients with the diagnosis of PAD on DUS. Demographic data, ABI, TBI, and DUS parameters of the dorsal pedis and lateral plantar arteries (DPA and LPA) were recorded.

**Results:**

Seventy-seven patients with 88 limbs were included, aged 69 [interquartile range: 11 years] with 28.6% of diabetic patients. The highest acceleration time of either DPA or LPA (AT^max^) was the most correlated to TBI on both univariate (*r* = −0.78, *p* < 0.0001) and multivariate analysis (*p* < 0.0001). DUS parameters had a weaker correlation with ABI. AT^max^ > 215 ms showed high diagnosis accuracy to a toe pressure of 30 mmHg or less [sensitivity of 86% [0.57–0.98] and negative predictive value of 97% [0.89–1.00]].

**Conclusion:**

AT^max^ demonstrates a high correlation with TBI in patients with PAD, and high diagnostic accuracy for detection of critical limb ischemia. Based on these results, AT^max^ can represent the next step in evaluating PAD severity with DUS, in patients with advanced lower extremity PAD.

## Introduction

Lower extremity peripheral artery disease (PAD) is a major burden worldwide, with a steady and significant increase in prevalence over the last decades (1, 2). PAD presents with various symptoms ranging from mild arterial claudication to critical limb ischemia, depending on different factors (3), including muscle's anaerobic functioning capacity, number, and efficiency of collateral pathways.

Ankle-brachial index (ABI) and toe-brachial index (TBI) are both accepted methods for diagnosing and assessing PAD severity, as a reflection of lower extremity perfusion (4). ABI is the most commonly used due to easy access, but has several limitations (5, 6). On the contrary, TBI is not limited by calcified arteries but the technique is less widely used because of longer acquisition and higher cost.

Arterial duplex ultrasound (DUS) has shown a good correlation with angiography on iliac and femoral arteries, but remained weak on distal arteries (7). Hence, the place of DUS with Doppler waveforms (DWs) analysis to estimate distal perfusion remains poorly known even if many consider it a helpful tool to evaluate PAD severity through distal perfusion. Recently, Sommerset et al. have emphasized the correlation of acceleration time of the lateral plantar artery (LPA) with ABI (8) and its relationship to critical limb ischemia (9). However, the relationship of the acceleration time with TBI is unknown.

Therefore, this study (i) evaluates the correlation between Doppler ultrasound of distal arteries of the lower extremities (dorsalis pedis artery and LPA) with TBI, in patients with occlusive PAD; (ii) evaluates the correlation between DW parameters of distal arteries of the lower extremities with ABI; and (iii) assesses the diagnostic accuracy of the highest acceleration time of pedal arteries to detect toe pressure ≤30 mmHg.

## Methods

### Patients Selection

A retrospective study was conducted between January 1 and December 31, 2020 in a single university hospital, from consecutive patients, addressed to our vascular medicine unit for diagnosis or follow-up of PAD.

Diagnosis of occlusive PAD was the only inclusion criterion. Diagnosis of PAD was based on the Doppler ultrasound criteria and is given below. For patients with occlusive PAD on both lower limbs, each limb was included separately.

Proximal occlusive PAD was defined as occlusion of at least one of the following arteries: aorta or common iliac artery, external iliac artery or common femoral artery, femoral superficial artery, or popliteal artery, diagnosed or confirmed by DUS. Patients with occluded iliac or femoral artery bypass were also included in the study. Patients with distal PAD (stenosis and/or occlusion of at least one of below-the-knee arteries) and/or proximal non-occlusive PAD (“significant stenosis, with no occlusion, on at least one artery from aorta to popliteal artery”) were not included.

Exclusion criteria were: age under 18 years old, toe or more proximal amputation, protected adults, history of arterial revascularization procedures on the included lower limb causing permeability of arterial axis for the aorta to the popliteal artery.

The following demographics data were obtained from the patient electronic medical record: age, gender, declared walking distance before pain, tobacco habits, arterial blood pressure, history of hypercholesterolemia, diabetes, and or severe to terminal chronic kidney failure (defined by a creatinine clearance of <30 ml/min/1.73 m^2^). For this study, patients were considered as a non-smoker if they had never smoked or declared at least 1-year tobacco cessation. Otherwise, they were considered active smokers.

After written information was sent by mail, no patient declared to be opposed to anonymously participating in this study. A lack of answer was considered as a non-opposition. Written consent from the participants was not required to participate in this study in accordance with the national legislation and the institutional requirements. Ethical review and approval were not required for this study on human participants because of its retrospective nature, in accordance with the local legislation and institutional requirements. This work was carried out accordingly to the Helsinki rules and local rules for a clinical study. It has been declared to the French National Commission for Liberties and Informatics (MR5914241120).

### Duplex Waveforms (DWs) Measurements

All measures were performed by vascular physicians (JET, MC, and CT with, respectively, 6, 3, and 20 years of experience).

**Figure 1:**
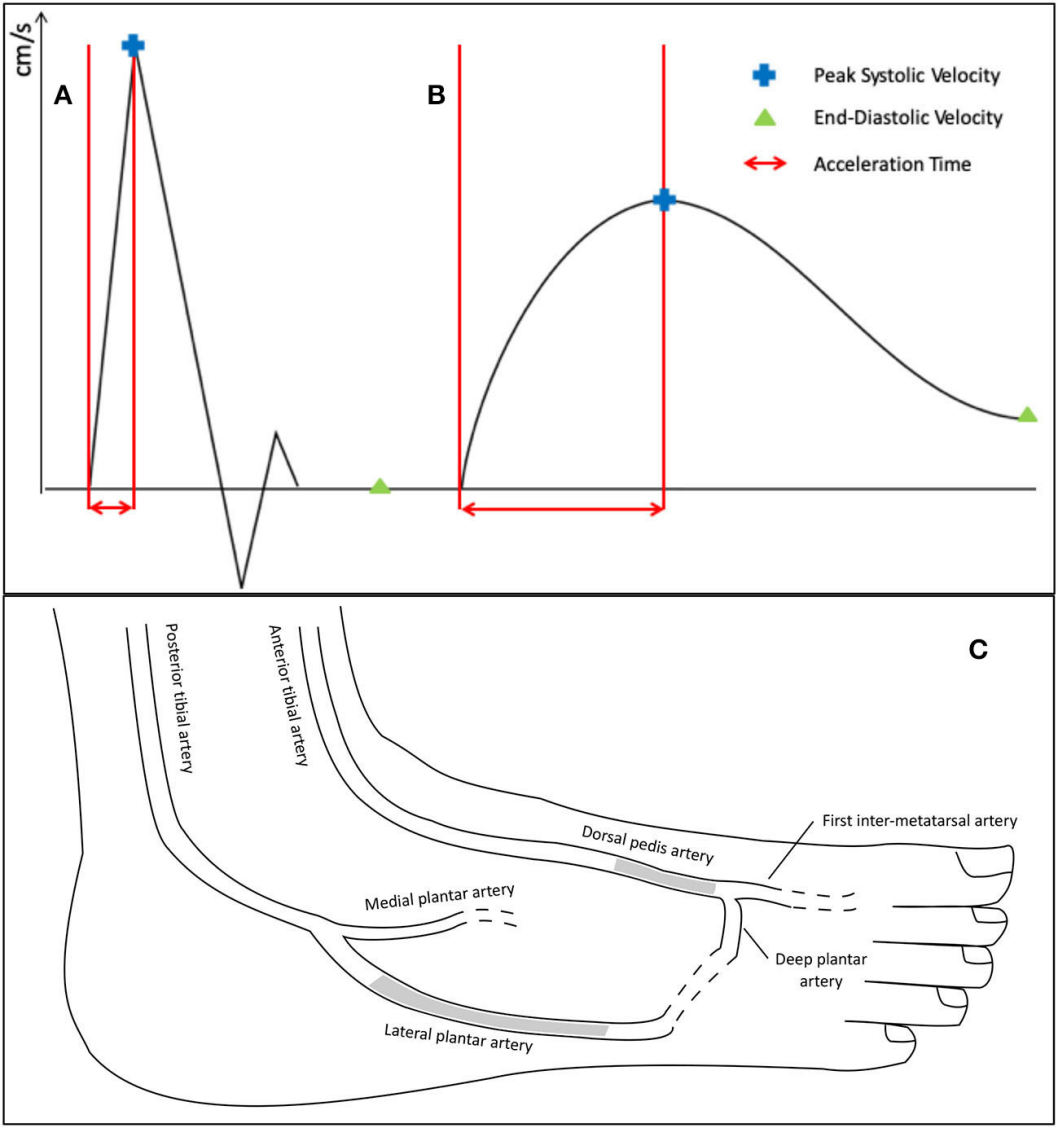
Technique and localization of duplex waveforms measurements of the dorsal pedis artery and lateral plantar artery. The **(A,B)** represent the parameters measured manually on different duplex waveforms: **(A)** corresponds to a triphasic waveform, classified N in the simplified Saint-Bonnet classification; and **(B)** is a monophasic attenuated (or “blunted”) waveform, classified as D-cf in the simplified Saint-Bonnet classification (10). Acceleration time is determined manually from the start of the systolic up-rise to the top of the systolic peak. **(C)** is an anatomic drawing of major pedal arteries. The grayed areas show the localization of the Doppler ultrasound recordings of the dorsal pedis artery and lateral plantar artery during the study.

Patients were scanned in decubitus position. Duplex Doppler ultrasound imaging was performed using a GE Vivid E95 (GE Healthcare Systems, Chicago, Illinois, USA) or a Siemens Sequoia (Siemens Healthineers AG, Erlanger, Germany). A 4–9 MHz linear array transducer was used for popliteal, fibular, anterior, and posterior tibial arteries, and a 5–14 MHz one for the dorsalis pedis artery (DPA) and LPA. Each ultrasound probe was covered with transmitting gel. Pulsed Doppler waveforms were obtained from a longitudinal slice of the studied artery, with a Doppler color box size adjusted to the size of the vessel. The emission sound beam was placed to be parallel to the flow direction of the vessel. The Doppler sample was obtained at 60° or less, with a volume applied to the center of each artery with a spectral Doppler scrolling of 66 mm/s. Peak-systolic velocity (PSV) and end-diastolic velocity (EDV) were measured manually and expressed in cm/s. Resistivity index (RI), without unit, was calculated as $\frac{PSV-EDV}{PSV}$. Acceleration time (AT, also called systolic rise time) was measured and expressed in milliseconds, after determining manually the start of the systolic uprise to the top of the systolic peak (8, 10) (Figure 1). Every study physician measured AT on three different DWs belonging to the same recording. The mean value of the three measurements was retained.

Two arteries were studied: DPA and LPA (Figure 1). DPA and LPA were considered occluded when a permanent absence of flow on Doppler color, and on pulsed Doppler in triplex mode, was observed. In case of a retrograde flow, the artery was considered patent, and Duplex waveforms were analyzed as usual.

Regarding proximal occlusive PAD, diagnosis of the occluded artery was retained when no flow was recorded on Doppler color and on pulsed Doppler using a triplex ultrasound test.

### Ankle-Brachial Index (ABI), Toe-Brachial Index (TBI), and Systolic Humeral Blood Pressure Measurements

All pressure measurements were systematically performed after DW measurements within 15 min.

Systolic humeral blood pressures were measured in both arms with an automatic device (Carescape Dinamap V100, GE Healthcare Systems, Chicago, Illinois, USA) with the patient in a supine position after at least 10-min of rest. Blood pressure cuffs were adapted to the weight and height of the patient (10-cm or 12-cm cuff).

Bilateral systolic blood pressure (SBP) on the dorsalis pedis artery and the posterior tibial artery was measured with an 8-MHz continuous Doppler probe (Diadop 200, Diatecnic, Labege, France) and a sphygmomanometer (Tycos TR-2 with 29–42 cm cuff, WelchAllyn, Skaneateles Falls, NY, USA or Lian Nano with 23–34 cm cuff, Spengler, Aix-En-Provence, France). If blood flow was still detected with a pressure cuff inflation of 250 mmHg or more, arteries were considered as “incompressible.” The value of 250 mmHg was considered as relevant as previously described in international recommendations (11), with higher pressure values being associated with potential bias due to pain of the patient.

Ankle-brachial index (ABI) was then calculated by dividing the highest value of the two SBPs of the leg by the highest value of the right and left arm SBPs (11).

Toe blood pressures were measured by a Laser Doppler flowmetry, using an automatic measure device (Periflux 6000 Combined, Perimed, Stockholm, Sweden), and TBI was calculated by dividing the toe pressure by the highest value of right and left arm SBPs. Pressure measurement was conducted according to the instructions of the manufacturer and as described in the literature (12). A pressure cuff (adapted to the toe size) was placed at the base of the big toe, and the laser Doppler probe (with the inflation cuff) at the top of the toe. The cuff was inflated at 200 mmHg and then was automatically and linearly deflated. SBP was read automatically by software when the LD probe detected the return of blood perfusion.

The performer of TBI was not blind to the DW measurements but due to automatic measuring of TBI, we consider the ranking bias to be minimal.

### Parameters Analysis

The following DW parameters were studied: PSV, EDV, RI, and AT on both DPA and LPA, the sum of PSV (from DPA and LPA), highest, lowest, and mean value of AT between DPA and LPA (AT^max^, AT^mean^, and AT^min^, respectively).

The PSV of an occluded artery was noted 0 cm/s and was recorded as such in the calculation of the sums of PSV of the distal arteries.

### Statistical Analysis

Our primary objective was to evaluate the correlation between Doppler ultrasound of distal arteries of the lower extremities (DPA and LPA) with TBI in patients with occlusive PAD, on both univariate and multivariate analyses. Age and ABI (as known marker/risk factor of PAD) were also compared to TBI.

Our second objective was to evaluate the diagnostic accuracy of the most TBI-correlated variable to detect toe pressure ≤30 mmHg.

Statistical analysis was conducted in R 4.0.2 software (R Foundation for Statistical Computing, Vienna, Austria; www.R-project.org), using the R studio interface. Because this cohort was not derived from random selection, all statistics are deemed to be descriptive. No imputation was made for missing data. Continuous variables are expressed as means and interquartile range (IQR), defined as the first and third quartiles. Categorical variables are expressed as counts and percentages. To evaluate the concordance of the Doppler measures, the correlation of DW parameters between contiguous arteries (e.g., anterior tibial artery and dorsalis pedis artery) was tested by Pearson's correlation. Pearson's correlation test was used to correlate Doppler waveform measures with TBI for univariate analysis. All quantitative variables had a normal distribution with no outliers, validating Pearson's correlation test. A Pearson's correlation >0.68 is considered a strong/excellent correlation (13).

For multivariate analysis, we used multiple linear regression. We kept as variables (a) known risk factors of PAD in literature (4) (tobacco use, hypertension, dyslipidemia, diabetes mellitus, history of stroke or myocardial infarction, and age) and (b) variables associated with TBI on univariate analysis with value of *p* < 0.2. Variables with high collinearity were excluded (variance inflation factor ≥5) (14).

Receiver operating characteristics (ROC) curve represented sensitivity vs. 1—specificity (i.e., true positive results plotted against false positive results) of the highest acceleration time of pedal arteries according to the presence or absence of toe pressure ≤30 mmHg; and was used to optimal cut-off value to detect toe pressure ≤30 mmHg. The area under the ROC curve (AUC) was used to measure the accuracy of the highest acceleration time of pedal arteries to detect toe pressure ≤30 mmHg. Significance was accepted for value of *p* < 0.05. No indeterminate results were recorded.

## Results

A total of 92 patients were screened. Six were excluded for the absence of toe pressure measurements, and nine were excluded for the absence of recording of either dorsal pedis artery or LPA. Seventy-seven patients were finally included with a total of 88 limbs.

Patient characteristics are summarized in Table 1. Most patients were male (77.3%). Forty patients (51.9%) were not exposed to tobacco at inclusion (never smoked or an at-least 1-year cessation). Median ABI was 0.63 [0.47–0.75]. As expected, 27 limbs (30.7%) had a TBI ≤0.3 and 14 (15.9%) had toe pressure ≤30 mmHg.

**Table 1:** Characteristics of the included patients (*n* = 77).

**Patient characteristics**	**Value (*n* = 77)**
Age (years; median [IQR])	69 [64–75]
Female sex (%)	20 (22.7)
**Treatment (%)**	
Platelets aggregation inhibitors	60 (77.9)
Statins	41 (53.2)
Antihypertensive	63 (81.8)
Hypertension (%)	64 (83.1)
Dyslipidemia (%)	50 (64.9)
Diabetes (%)	22 (28.6)
Chronic kidney disease (<30 mL/min/1.73 m^2^) (%)	11 (14.3)
Tobacco users (%)	37 (48.1)
Tobacco use [pack-year, median (IQR)]	50 [40–80]
History of myocardial infarction and/or stroke (%)	20 (26.0)
Declared walking distance (%)	300 [150–457.5]
**Limb characteristics **	**Value (*****n*** **=** **88)**
**Level of occlusion %**	
Aorta/common iliac artery	14 (15.9)
External iliac artery/common femoral artery	5 (5.7)
Superficial femoral artery/popliteal artery	69 (78.4)
**Dorsalis pedis artery**	
Occluded artery (%)	19 (21.6)
Peak systolic velocity [cm/s; median (IQR)]	14.0 [9.2–24.1]
End diastolic velocity [cm/s; median (IQR)]	4.2 [1.4–7.7]
Resistivity index [no unit; median (IQR)]	0.65 [0.49–0.87]
Acceleration time [milliseconds; median (IQR)]	170 [141–209]
**Lateral plantar artery**	
Occluded artery (%)	20 (22.7)
Peak systolic velocity [cm/s; median (IQR)]	13.7 [9.6–20.3]
End diastolic velocity [cm/s; median (IQR)]	3.9 [1.6–6.7]
Resistivity index [no unit; median (IQR)]	0.72 [0.56–0.84]
Acceleration time [milliseconds; median (IQR)]	160 [134.1–200]
Ankle brachial index [no unit; median (IQR)]	0.63 [0.47–0.75]
Highest ankle pressure [mmHg, median (IQR)]	90 [65–105]
**Toe brachial index [no unit; median (IQR)**]	0.40 [0.27–0.50]
≤0.3 (%)	27 (30.7)
[0.3–0.5] (%)	41 (46.6)
[0.5–0.7] (%)	18 (20.5)
≥0.7 (%)	2 (2.3)
Toe pressure ≤30 mmHg (%)	14 (15.9)

*IQR, interquartile range*.

Table 2 summarizes the linear correlation coefficients (using univariate analysis) between (a) DW measurements on DPA and LPA and (b) both TBI and ABI. Acceleration time (AT) on both DPA and LPA showed a very strong inverse correlation with TBI (*r* = −0.75 and −0.75, respectively).

**Table 2:** Correlation between different pulsed Doppler measures, ankle-brachial index, and toe-brachial index using univariate analysis.

**Variable**	**Pearson's correlation test**			
	**TBI**		**ABI**	
	**Coefficient (*r*)**	***p*-value**	**Coefficient (*r*)**	***p*-value**
Age	0.03	>0.2	0.06	>0.2
ABI (ankle-brachial index)	0.53	<0.001	–	–
**Dorsalis pedis artery (DPA)**				
PSV (peak systolic velocity)	0.11	>0.2	0.14	>0.2
EDV (end-diastolic velocity)	−0.25	>0.2	−0.16	>0.2
RI (resistivity Index)	0.63	<0.001	0.42	<0.01
AT (acceleration time)	−0.75	<0.001	−0.44	<0.01
**Lateral plantar artery (LPA)**				
PSV (peak systolic velocity)	0.26	>0.2	0.13	>0.2
EDV (end-diastolic velocity)	−0.25	>0.2	−0.23	>0.2
RI (resistivity Index)	0.63	<0.001	0.51	<0.001
AT (acceleration time)	−0.75	<0.001	−0.50	<0.001
**Doppler indexes**				
Sum of PSV (DPA/LPA)	−0.05	>0.2	0.06	>0.2
Sum of AT (DPA/LPA)	−0.75	<0.001	−0.41	<0.01
AT^max^	−0.78	<0.001	−0.46	<0.01
AT^mean^	−0.75	<0.001	−0.41	<0.01
AT^min^	−0.77	<0.001	−0.48	<0.001

*ABI, ankle-brachial index; AT, acceleration time; AT^max^, highest acceleration time between pedal arteries; AT^mean^, mean value of acceleration time between pedal arteries; AT^min^, lowest acceleration time between pedal arteries; DPA, dorsalis pedis artery; DW, duplex waveform; EDV, end-diastolic velocity; PSV, peak systolic velocity; TBI, toe-brachial index*.

**Figure 2:**
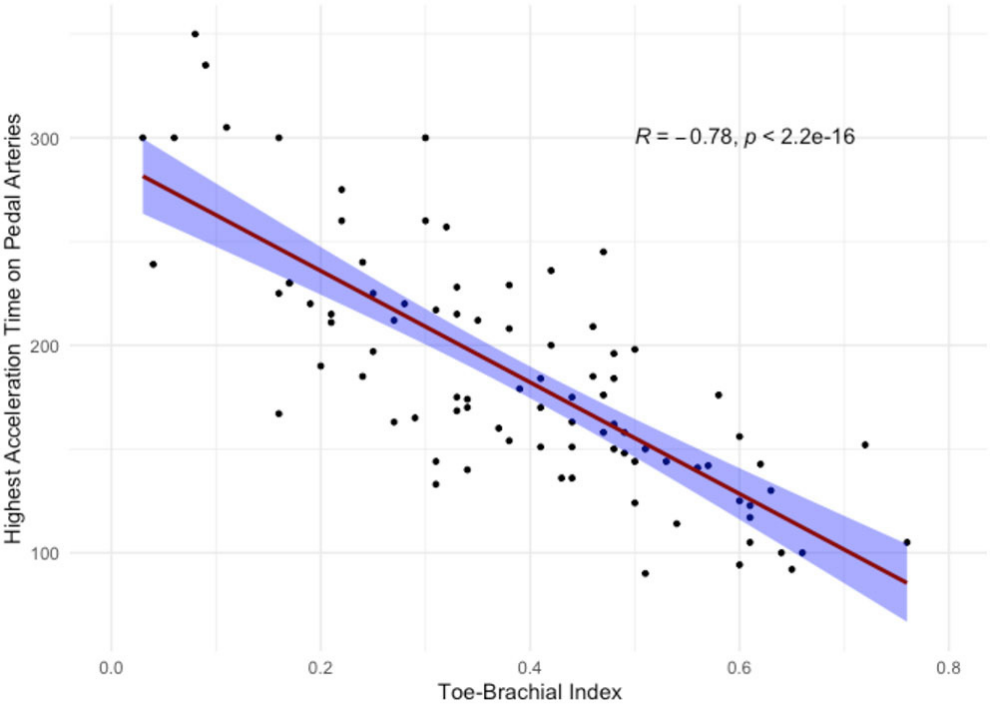
Scatterplot and linear correlation of toe-brachial index with highest value of acceleration time between dorsalis pedis artery and lateral plantar artery. Correlation straight line is red-colored with blue-filled standard errors. *R*, Pearson's correlation coefficient; *p, p*-value.

The highest correlation to TBI comes about with the highest value of AT between DPA and LPA (*r* = −0.78). Figure 2 shows the scatterplot and linear correlation between TBI and the highest value of AT (DPA or LPA).

AT^max^ remained strongly associated with TBI on multivariate analysis (*p* < 0.0001) (Table 3).

**Table 3:** Multiple linear regression model outputs comparing duplex waveform parameters and known risk factors to toe-brachial index.

**Variables**	**Value**	**Std. error**	***p*-value**
AT^max^	−0.0019	0.0004	7.4 × 10^−4^
Diabetes	0.045	0.038	0.23
Male sex	−0.429	0.040	0.29
ABI	0.074	0.072	0.31
Active tobacco use^†^	0.024	0.030	0.43
Chronic kidney failure	0.040	0.055	0.47
Resistivity index of LPA^††^	−0.091	0.232	0.48
Dyslipidemia	−0.016	0.032	0.62
History of stroke / MI	0.016	0.034	0.65
Resistivity index of DPA^††^	−0.012	0.137	0.93

*As expected, acceleration time of DPA and LPA, AT^mean^ and AT^min^ showed very high collinearity and were excluded*.

*ABI, ankle-brachial index;AT, acceleration time; AT^max^, highest acceleration time between pedal arteries; DPA, dorsalis pedis artery; DW, duplex waveform; MI, myocardial infarction*.

† *Active tobacco use was defined by active smokers or tobacco cessation for <1 year*.

† † *Results of resistivity index on DPA and LPA must be interpreted with caution because both variables showed moderate but significant collinearity (VIF = 7.1 and 8.4, respectively)*.

Regarding our second objective, every DW parameter significantly correlated to TBI was also to ABI but correlation coefficients were systematically lower.

**Figure 3:**
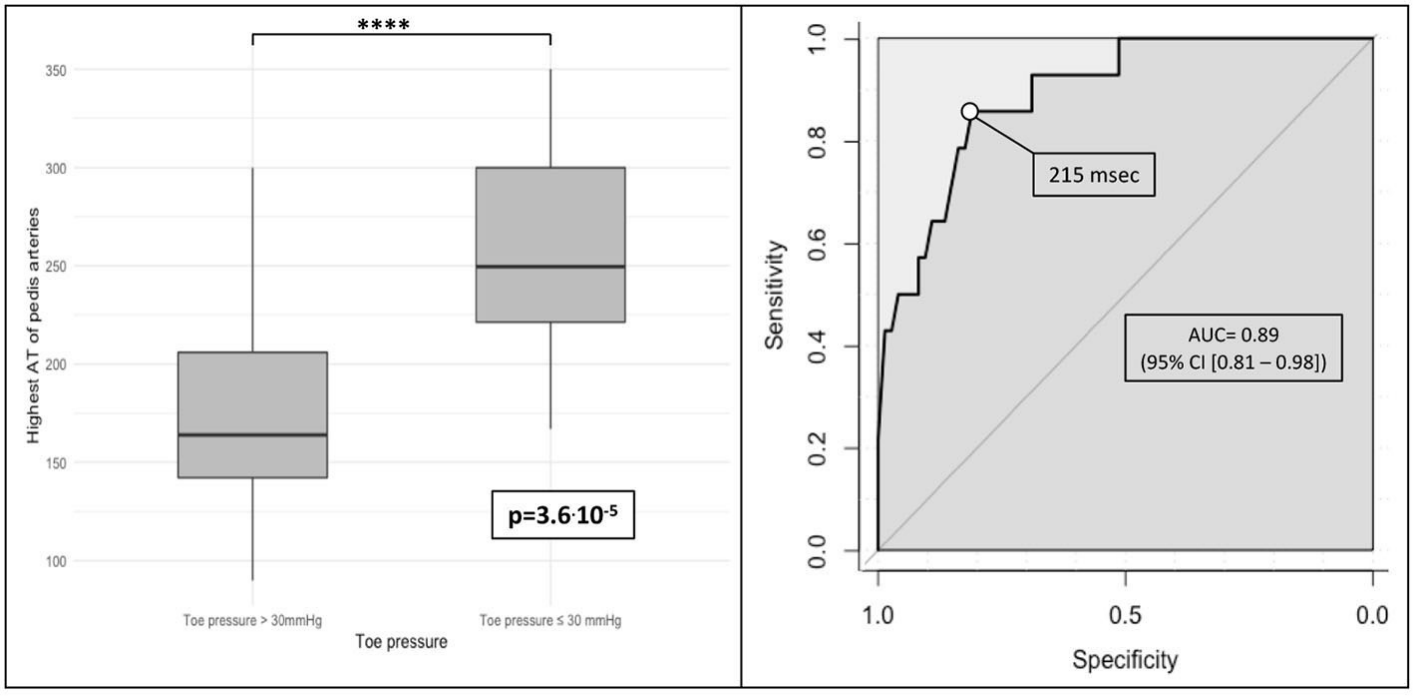
Diagnosis accuracy of highest acceleration time of pedal arteries (AT^max^) to detect toe pressure ≤30 mmHg (boxplot and area under ROC curve). ROC, receiver operating characteristic; msec, milliseconds; *p, p*-value; ns, non significant. *p < 0.05, *p < 0.01, **p < 0.001, ***p < 0.0001, ****p < 0.00001.

Regarding our third objective, we analyzed the diagnosis accuracy of the highest AT of pedal arteries to detect toe pressure ≤30 mmHg. Figure 3 shows that the highest AT of pedal arteries (AT^max^) statistically differs according to the presence or absence of toe pressure ≤30 mmHg: 171.4 vs. 259.7 ms, respectively, *p* < 0.0001). Diagnosis accuracy of AT^max^ is high with an area under curve of 0.89 [95% CI [0.81–0.98]] (Figure 3). According to ROC curve analysis, the optimal cut-off value was 215 ms. Table 4 summarizes the diagnostic performances of AT^max^ with the cut-off value of ≥215 ms.

**Table 4:** Diagnostic performances of ATmax to diagnose toe pressure ≤30 mmHg.

**Test**	**Value^**a**^**
Cut-off value	≥215 ms
Sensitivity	85.7 (57.2–98.2)
Specificity	81.1 (70.3–89.3)
Positive predictive value	46.2 (26.6–66.7)
Negative predictive value	96.8 (88.8–99.6)
Positive likelihood ratio	4.53 (2.70–7.60)
Negative likelihood ratio	0.18 (0.05–0.64)
Area under ROC curve	0.89 (0.81–0.98)
Youden index	66.8 (27.5–87.5)
Diagnostic odds-ratio	25.7 (5.2–128.1)

a *Results are expressed with 95% confidence interval*.

## Discussion

Most international consensus recommends evaluation and quantification of PAD through clinical factors (ABI, walking distance, and rest pain) and imaging, such as CT angiogram, magnetic resonance angiogram, and DUS (4, 15–17). Localization and grading of arterial stenosis with DUS have already been studied with a good sensitivity and specificity (18–21). Nevertheless, the use of DUS to estimate the global severity of PAD is scarce in the literature. This study demonstrates that the highest value of AT^max^ of either DPA or LPA is highly correlated to TBI and can be used to diagnose toe pressure lower or equal to 30 mmHg, one of the criteria used to defined critical limb ischemia (CLI) (22). AT^max^ with a cutoff value of 215 mmHg has high diagnostic accuracy: a sensitivity equal to 86%, a specificity of 81%, and a negative predictive value of 97%.

Acceleration time seems to be more and more described in the literature as associated with PAD. For example, in 2020, Yagyu et al. published that the acceleration time ratio between the popliteal (or distal superficial femoral artery) and the common femoral artery was highly predictive of femoropopliteal artery lesions with an AUC of 0.93 (23). Recently, Sommerset et al. described plantar acceleration time (PAT) as a novel predictor of PAD. In non-diabetic patients, PAT was highly correlated to ABI (8, 24). However, ABI is a challenging tool to detect and quantify PAD especially in patients with multiple comorbidities (diabetes, chronic kidney failure, and advanced age) and/or severe PAD (5, 6). For this “challenging” population, TBI shows far better sensitivity (25) but the relationship between TBI and AT has never been studied. The main strength of our study is to propose a more accessible diagnostic tool in patients with occlusive PAD, especially when TBI is not accessible for the practitioner.

In our study with patients with occlusive PAD, no DW variable showed a high correlation with ABI. This result is understandable considering the low sensibility of ABI in this population (5, 6, 25). Despite the contradictory statement in literature, these results tend to favor the use of TBI in a “challenging population” (26).

Our results are not consistent with those of Sommerset et al. (8) as, in our study, PAT was well-correlated to TBI but was not associated with ABI. To our point of view, this result does not reflect the lack of sensitivity of PAT for diagnosis of severe PAD but more the limitation of the use of ABI in these patients. PAT was not the only variable well-correlated to TBI; indeed, an acceleration time of the DPA showed just as satisfying results (*r* = −0.75 and −0.75, respectively). This led us to the conclusion that the highest value of acceleration time of either pedal artery (AT^max^) allowed us to moderately increase the correlation (*r* = −0.78) but also to have a satisfying measure for every included patient. Considering PAT or DAT, missing values were recorded in 22.7 and 21.6% of our population, respectively, due to arterial occlusion. These results could perhaps be explained by the fact that our included patients had more severe PAD. When we considered the highest acceleration time, AT^max^ could be measured in all our patients as none presented concomitant dorsal and plantar arterial occlusion. On multivariable analysis (with known risk factors and variables with the highest correlation on the univariate analysis), AT^max^ remained highly associated with TBI (*p* < 0.001).

To help categorize PAD severity through DW, recent studies have studied the benefit and reproducibility of a 13-category arterial Doppler waveform classification, called the “Saint Bonnet classification” (10, 27).

Even if the highest value of acceleration time on pedal arteries showed the best correlation to TBI, mean (AT^mean^) and lowest (AT^min^) acceleration time on pedal arteries show strong correlations with TBI. These results need to be replicated on the wider population to confirm the superiority of AT^max^ compared to AT^mean^ and AT^min^.

More recently, Teso et al. showed that change of pedal acceleration time between pre- and post-revascularization surgery is associated with limb salvage in patients with noncompressible ABIs (9). However, it is unclear which acceleration time determined the “pedal acceleration time,” as they recorded duplex waveforms from the arcuate artery, the first dorsal metatarsal artery, and the lateral, medial, and deep plantar arteries.

Based on our results, AT^max^ is a diagnosis that helps in patients with occlusive PAD. AT^max^ showed high sensitivity and negative predictive value to detect toe pressure ≤30 mmHg, one of the criteria for critical limb ischemia in literature. The optimal cut-off value was 215 ms in our study, which is concordant with the results of Sommerset et al., who considered PAT >225 ms to be associated with severe PAD and CLI (8).

### Limitations

Several limitations in this study should be addressed. First, the study was retrospective which cannot provide exhaustive bias control. The number of included patients is modest (*n* = 88). However, this study was a proof of concept study and this preliminary result should be validated by a prospective study including a larger number of patients. Second, the prognosis value of AT^max^ was not evaluated in this work since no patient follow-up was performed. This was not the aim of the study but further studies are required. Third, all ABI and TBI measures were performed by three vascular physicians and intraindividual variability was not evaluated. However, these measures were performed by highly trained and experienced vascular physicians. Moreover, TBI reproducibility could be considered satisfactory due to automatic measuring.

Last, interindividual variability and intraindividual reproducibility were not assessed in this study but should be studied before the large-scale use of this new predictive tool.

### Future Direction

Before a large-scale use, AT^max^ needs to be validated in an external population and studied on a broader patient population. Moreover, a thorough assessment of the intraindividual reproducibility and interindividual variability is paramount for the generalization of this new tool.

## Conclusion

This study suggests that the highest acceleration time of either DPA or LPA (AT^max^) is highly correlated to TBI in patients with occlusive PAD. AT^max^ with a cut-off value of 215 ms could represent a step forward for the diagnosis of toe pressure ≤30 mmHg, one of the criteria of critical limb ischemia. This measure might be of great interest to clinicians who do not have access to TBI.

More studies are needed to confirm if the highest acceleration time of pedal arteries is more predictive of PAD severity than plantar acceleration time alone; and confirm the optimal cut-off value for detecting critical limb ischemia.

## Data Availability Statement

The raw data supporting the conclusions of this article will be made available by the authors, without undue reservation.

## Ethics Statement

Ethical review and approval was not required for the study on human participants in accordance with the local legislation and institutional requirements. Written informed consent for participation was not required for this study in accordance with the national legislation and the institutional requirements.

## Author Contributions

J-ET, GM, MC, CT, VC, JG, and DL: conception and design, revising, approval for publication, and agree to be accountable for all aspects of the work. J-ET, GM, JG, DL, and CT: data analysis. J-ET, GM, DL, and MC: drafting. All authors contributed to the article and approved the submitted version.

## Conflict of Interest

The authors declare that the research was conducted in the absence of any commercial or financial relationships that could be construed as a potential conflict of interest.

## Publisher's Note

All claims expressed in this article are solely those of the authors and do not necessarily represent those of their affiliated organizations, or those of the publisher, the editors and the reviewers. Any product that may be evaluated in this article, or claim that may be made by its manufacturer, is not guaranteed or endorsed by the publisher.
